# Case Report: Advanced pulmonary sarcomatoid carcinoma with adrenal gland metastasis after sintilimab combined with anlotinib treatment

**DOI:** 10.3389/fonc.2023.1167516

**Published:** 2023-06-30

**Authors:** Gangyi Dai, Lang He, Qin Yan, Yamao Li, Yuandong Huang, Bin Li, Guoping Wang

**Affiliations:** ^1^ Department of Oncology, the Chengdu Fifth People’s Hospital, Chengdu, Sichuan, China; ^2^ Affiliated Chengdu Fifth People’s Hospital of Chengdu University of Traditional Chinese Medicine (Second Clinical Medical School), Chengdu Institute of Cancer Prevention and Control, Chengdu, Sichuan, China

**Keywords:** case report, pulmonary sarcomatoid carcinoma, immunotherapy, metastasis, antiangiogenic therapy

## Abstract

**Background:**

Pulmonary sarcomatoid carcinoma (PSC) is a rare subtype of non-small-cell lung cancer (NSCLC), which is resistant to conventional chemotherapy and radiotherapy with a poor prognosis. The MET inhibitor may be effective for the patients with MET exon 14 skipping mutation. This mutation was not detected in this patient. However, the PD-L1 TPS 60%, KRAS and TP53 mutations were detected in this patient could benefited from immunotherapy. The anlotinib is a novel multitarget antiangiogenic drug that could be effective for advanced non-small-cell lung cancer and some sarcoma patients. We report a patient with advanced pulmonary sarcomatoid carcinoma successfully treated with immunotherapy combined with antiangiogenic drugs.

**Case summary:**

A 75-year-old male was admitted to the hospital in July 2020 because of productive cough for more than three months. The patient was diagnosed with advanced pulmonary sarcomatoid carcinoma with adrenal gland metastasis (cT4N3M1b, stage IVA) was treated in our hospital. Genetic testing revealed KRAS P.L19F mutation (abundance 19.12%) and NFEE2L2 P.E82G mutation (abundance 14.84%); TP53 P.S183 mutation (abundance 26.97%), TMB(Tumor Mutational Burden) 30.91 muts/Mb, MSS, and PD-L1 (Daco 22C3) TPS 60% were also detected. We administrated sintilimab combined with anlotinib treatment, a PD-1 inhibitor with antiangiogenic drug. The patient achieved a favorable outcome with tolerable adverse effects.

**Conclusion:**

Sintilimab combined with anlotinib treatment may lead to a favorable outcome for patients with advanced pulmonary sarcomatoid carcinoma.

## Introduction

Pulmonary sarcomatoid carcinoma (PSC) accounts for approximately 0.1%–0.5% of lung malignant tumors (1). The histological studies suggest that PSC is a group of transformed carcinoma that originates from the same primitive epithelium and forms after epithelial-mesenchymal transition (EMT). It is a hallmark of dedifferentiation in a progressing epithelial tumor, and this transition is a process that results in the co-existence of epithelial and stromal components in varying proportions. PSC exhibits features of both epithelial and mesenchymal tumors and is categorized as a subtype of non-small cell lung cancer (NSCLC) (2). In 2021, the World Health Organization (WHO) classified PSC into five subtypes on the basis of pathomorphological features, including pleomorphic carcinoma, spindle cell carcinoma, giant cell carcinoma, carcinosarcoma, and pulmonary blastoma (3).

The prognosis of PSC is worse than other subtypes of NSCLC, and the risk of death is 1.6 times higher than that of other NSCLCs (4). Traditional treatment and chemotherapy show limited efficacy for PSC, leading to a poor prognosis (5). The treatment options for advanced-stage patients are limited, and reports of the benefit of immunotherapy and targeted therapy in PSC are very rare.

The incidence of MET (Mesenchymal-Epithelial Transition Factor) exon 14 skipping mutations in PSC can be as high as 4.9%-31.8% (6). Patients with this mutation may benefit from MET inhibitor drugs such as Capmatinib and Tepotinib. However, no MET exon 14 skipping mutation was detected in this case. Currently, in some tumors such as lung cancer, higher expression of PD-L1 indicates a better response to immunotherapy. In lung cancer patients carrying KRAS (Kirsten ratsarcomaviral oncogene homolog)/TP53 (Tumour Protein 53) mutations, the expression of PD-L1 is significantly increased, leading to enhanced T-cell infiltration and immune sensitivity of the tumor. Therefore, patients with KRAS/TP53 mutations may benefit from PD-L1 inhibitors, although the overall survival rate varies greatly among different cancer types.

In this report, the patient with high PD-L1 expression and KRAS combined with TP53 mutation could benefit from immunotherapy. Anlotinib is effective in advanced non-small cell lung cancer and some sarcomas. Here we report a case of pulmonary sarcomatoid carcinoma (spindle cell carcinoma) diagnosed by CT(Computed Tomography)-guided percutaneous lung biopsy and successfully treated with sintilimab combined with anlotinib.

## Case presentation

### Chief complaints

A 75-year-old male was admitted to the hospital in July 2020 because of productive cough for more than three months.

### History of present illness

**Figure 1:**
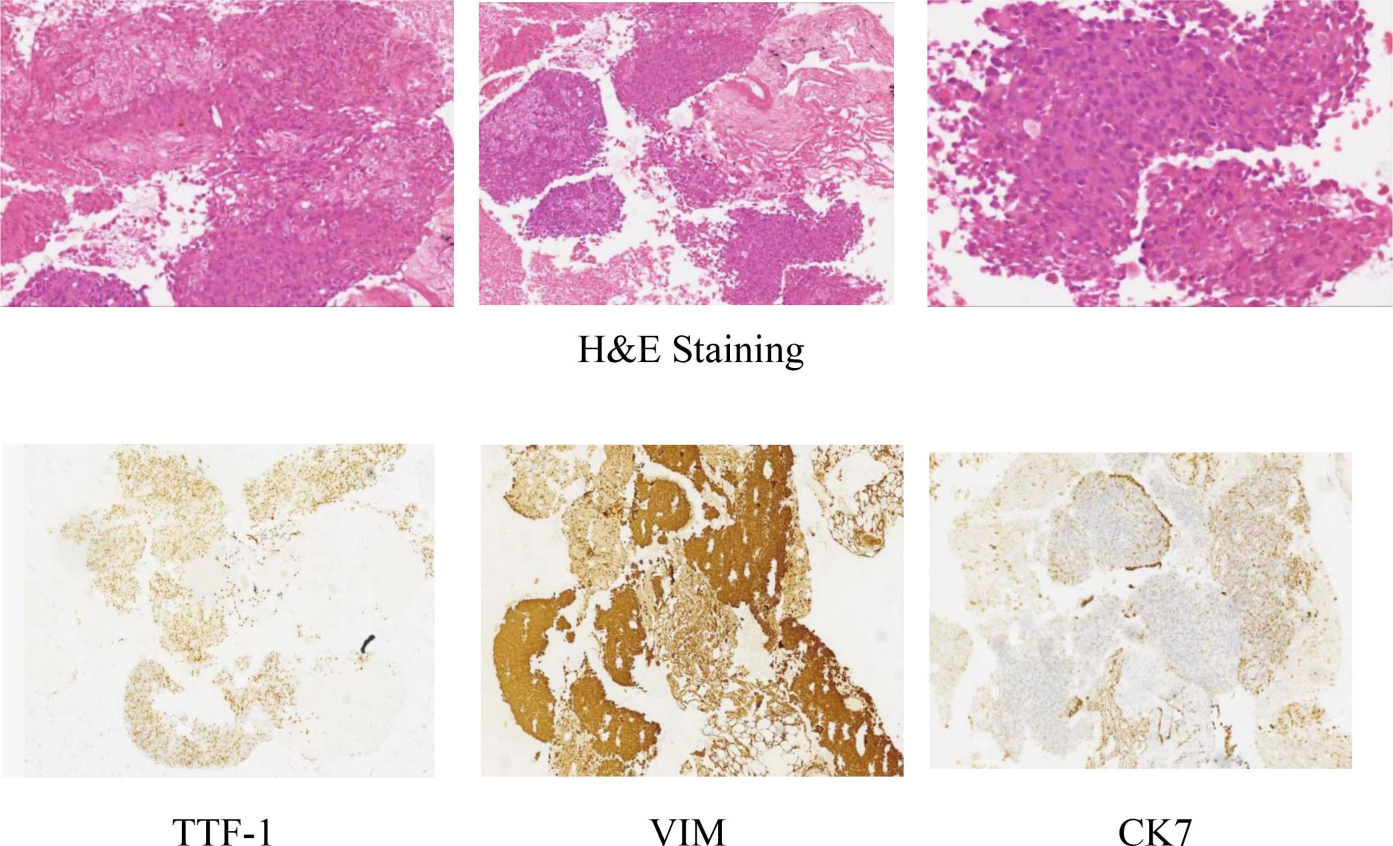
Pathology results of the biopsy.

On July 24, 2020, the patient underwent an enhanced CT scan of the chest, abdomen, and pelvis, which showed a mass in the lower lobe of the right lung and a multisystem neoplastic lesion. The left adrenal mass was considered as a metastatic tumor. The CT-guided lung biopsy was performed on July 28, 2020. Microscopically, it showed the tumors were composed of spindle cells. Immunohistochemically, it showed TTF1(+), CK(+), Vimentin(+), CK7(focal+), EGFR(2+), CD56(-), NapsinA(-), P63(-), P40(-), Ki67(+, about 70%). The features were consistent with pulmonary sarcomatoid carcinoma (spindle cell carcinoma) (**Figure 1**).

### History of past illness

The patient had level 2 hypotension for more than 20 years; he was treated with amlodipine 5 mg QD.

### Personal and family history

The patient had a smoking history of 40 cigarettes/day for more than 50 years. No information for family history.

### Physical examination upon admission

Height 165cm,Weight 60kg, ECOG(Eastern Cooperative Oncology Group) 1, NRS(Numerical Rating Scale) 2. No significant abnormal findings were found on physical examination.

### Laboratory examinations

On July 28, 2022, NGS (Next-generation Sequencing) testing for 520 target genes was performed. The results revealed that foci showed KRAS P.L19F mutation (abundance 19.12%) and NFE2L2 (Nuclear Factor Erythroid-derived 2-like 2) P.E82G mutation (abundance 14.84%); TP53 P.S183 mutation (abundance 26.97%), TMB (Tumor Mutational Burden) 30.91 muts/Mb, MSS (MicroSatellite Stability), and PD-L1 (Daco 22C3) TPS(Tumor cell Proportion Score)60% were detected.

Liver function, renal function, routine blood test, and infectious disease screenings revealed no significant abnormalities.

### Imaging examinations

**Figure 2:**
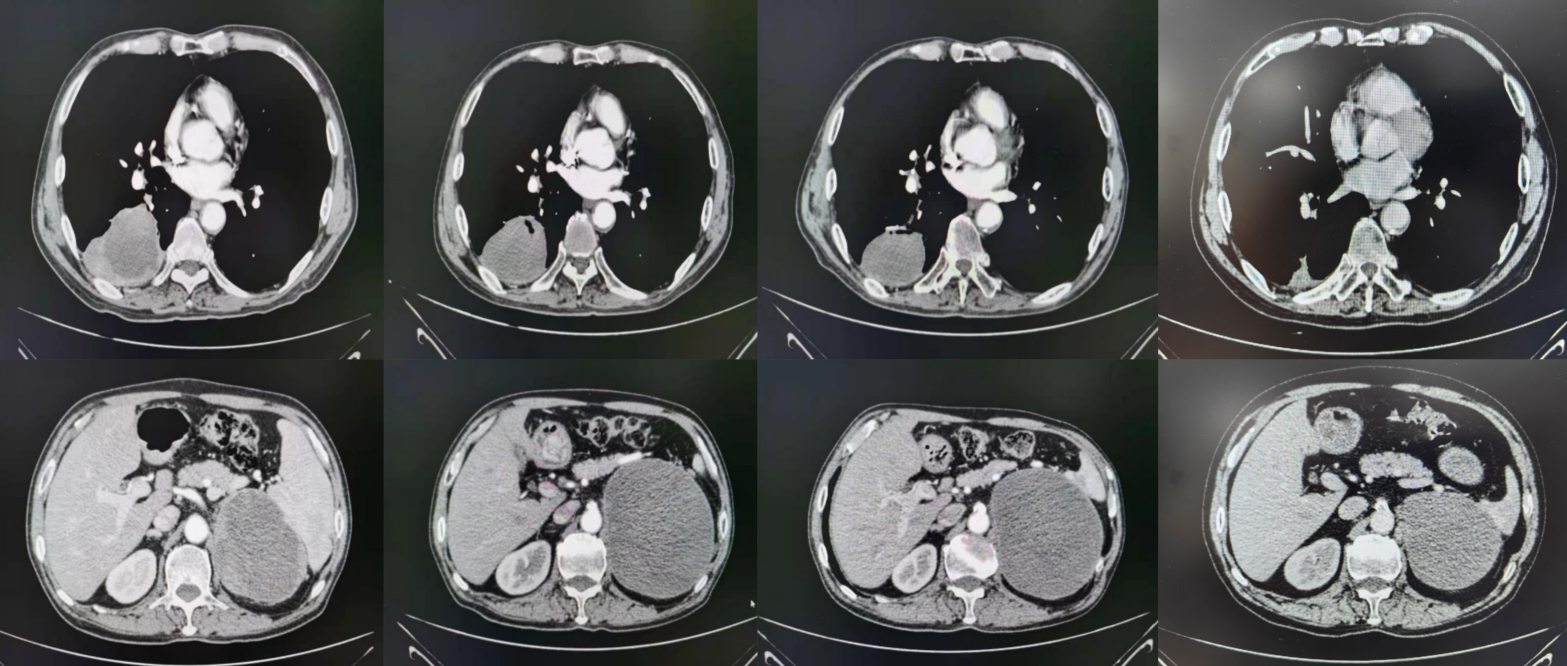
CT scan results of the patient.

On July 21, 2020, the patient underwent enhanced CT/MRI/SPECT scans. The results showed a mass in the right lower lobe of the lung, with a maximum size of 7.1 cm × 7.3 cm, and a mass in the left adrenal gland, with a maximum size of 10.2 cm × 7.5 cm. No obvious abnormalities were observed in head MRI(Magnetic Resonance Imaging)and SPECT(Single Photon Emission Computed Tomography). After treatments, the lesion of the lung was substantially reduced in size (**Figure 2**, **Table 1**).

**Table 1:** The size change of the lesions*.

Loci	Baseline (July 21, 2020)	November 26, 2020	February 27, 2021	March 16, 2023
Right Lung	71*73mm	67*57mm	61*45mm	25*18mm
Left adrenal gland	102*75mm	100*120mm	104*122mm	98*84mm

*Treatment: anlotinib 12 mg QD from day 1 to 14 combined with sintilimab 200 mg on day 1, q3w*17 cycles.

**Figure 3:**
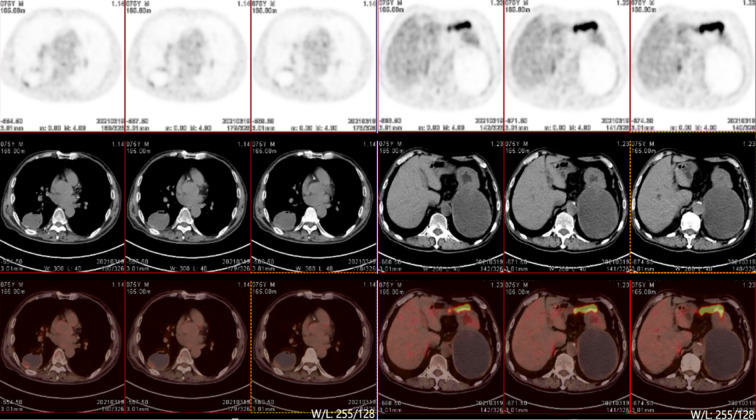
PET/CT scan results of the patient.

On March 19, after 7 months of treatment, PET-CT(Positron Emission Tomography -Computed Tomography) examination was performed and showed that the round cystic solid density shadow in the dorsal segment of the right lower lobe showed a slight increase in 18F-FDG(18F-fluorodeoxyglucose) metabolism in the posterior margin wall (maximum SUV[standard uptake value] 1.9, mean value 1.4), suggesting that the local tumor activity was inhibited. The left adrenal gland showed round cystic solid hypodensity with slightly increased 18-FDG metabolism in the cyst wall (maximum SUV 0.9, mean SUV 0.7), suggesting that the local tumor activity was inhibited. No signs of metastasis were found (**Figure 3**).

### Diagnosis

The patient was diagnosed with sarcomatoid carcinoma of the right lower lobe of the lung with metastasis to the left adrenal gland (cT4N3M1b, stage IVA).

### Treatment

From August 2020 to June 2022, the patient received anlotinib 12 mg QD from day 1 to 14 combined with sintilimab 200 mg on day 1, q3w*17 cycles. The patient was treated at the Fifth People’s Hospital of Chengdu.

### Outcome and follow-up

The patient was underwent regular evaluations with chest-abdomen-pelvis enhanced CT, cranial-enhanced MRI, SPECT, and other examinations every 3 months roughly. The latest visit to our hospital was on March 16, 2023(9 months after treatments), during which the chest-abdomen-pelvis enhanced CT revealed a soft tissue mass in the right lower lobe of approximately 25*18mm, and a circular low-density shadow in the left adrenal area of approximately 98*84*80mm. The imaging indicated a significant reduction in the size of the right lung lesion and a slight reduction in the left adrenal lesion.

The treatment was generally successful, and no notable discomfort was reported. At present, the ECOG score of the patient is grade 1 without obvious adverse effects.

## Discussion

PSC is a rare subtype of non-small cell lung cancer (NSCLC) characterized by poorly differentiated tumor with sarcomatous or sarcomatoid components, rapid proliferation, high vascular invasion, and epithelial-mesenchymal transition. The median overall survival time (OS) is about 7 months (7). The incidence of MET 14 exon skipping mutation in patients with pulmonary sarcomatoid carcinoma is 5-32% (8), and patients with MET 14 exon skipping mutation could be treated by MET inhibitors effectively. However, the MET 14 exon skipping mutation was not detected in this patient. Immune checkpoint inhibitors can improve the prognosis of advanced non-small cell lung cancer. Compared with other types of non-small cell lung cancer, the expression level of PD-L1 in patients with pulmonary sarcomatoid carcinoma is higher, and a higher proportion of patients show high TMB. Therefore, pulmonary sarcomatoid carcinoma is expected to benefit from immunotherapy. Anlotinib as a novel multitarget antiangiogenic has been approved for the third-line treatment of advanced NSCLC by the Chinese Food and Drug Administration.

The NGS results for the patient in this study revealed KRAS and TP53 gene mutations, which are shown to be associated with benefit from immunotherapy (9). The MET 14 skipping mutation was not found. In a study in 2017, 15867 NSCLC patients, including 125 patients with PSC, were prospectively analyzed using the whole genome sequencing (CGP) platform (10). In PSC patients, the median number of gene variants per tumor detected by CGP was 5, and 99% of tumors showed at least one gene mutation. The most common mutations were in the TP53 (73.6%), CDKN2A(Cyclin-Dependent Kinase Inhibitor 2A) (37.6%), KRAS (34.4%), CDKN2B(Cyclin-Dependent Kinase Inhibitor 2B) (23.2%), and NF1(Neurofibromin 1) (17.6%) genes. Previous studies reported that the incidence of MET exon 14 skipping mutations in PSC is as high as 3%–31.8% (11).

Immune checkpoint inhibitors can improve the prognosis of patients with advanced NSCLC. However, whether PSC benefits from immunotherapy and the specific immunology features of PSC was still largely uncertain. Vieira et al. reported that among 75 PSC patients, 40 (53%) showed positive PD-L1 expression (12). Additionally, the proportions of CD3+ tumor-infiltrating lymphocytes and CD163+ tumor-associated macrophages were more abundant in PSC than in other subtypes of NSCLC. This finding suggests that PD-L1 expression is higher, with a higher proportion of CD3+ T cells and macrophage infiltration, in patients with PSC than in patients with other subtypes of NSCLC. This suggests that PSC patients may benefit from treatment with PD-1/PD-L1.

The current patient had high TMB (30.91 mut/Mb). In a previous study of 125 PSC patients (13), the median TMB was 8.1 mut/Mb (mean value 13.6, range 0–165.2); approximately 20% of patients had TMB > 20 mut/Mb and 43% of patients had TMB > 10 mut/Mb.

A real-world retrospective study revealed that PSC commonly harbors either potentially targetable genomic alterations or high TMB as observed by comprehensive genomic profiling (10). One study analyzed 37 PSC patients in France treated with immunotherapy. All patients had received platinum-based chemotherapy before immunotherapy, with a median treatment duration of four cycles. Nivolumab was the most frequently used treatment, with a median duration of 10 cycles (1–32 cycles). The objective response rate of immunotherapy was 40.5% (15/37), and the disease control rate was 64.8% (24/37). The median progression-free survival of patients was 4.89 months (range: 0.3–35.7 months), and the 1-year PFS (Progress Free Survival) rate was 15%. The median OS of patients was 12.7 months (range: 0.3–45.7), and the 1-year survival rate was 51.3% (14).

In this case, CT-guided percutaneous lung biopsy was performed. Microscopically, it showed the specimen was almost all spindle cells. Immunohistochemically, it showed the epithelial-related markers TTF-1 (+), CK (+), CK7 (focal+), and the mesenchymal-related marker Vimentin (+). Therefore, the final pathological report diagnosed pulmonary sarcomatoid carcinoma(spindle cell carcinoma).The current patient received anlotinib administered with sintilimab. The VEGFR(Vascular Endothelial Growth Factor Receptor) signaling pathway regulates the immune response by reducing tumor T cell infiltration and increasing immune suppressor cells, such as regulatory cells and myeloid-derived suppressor cells, ultimately leading to an immunosuppressive tumor microenvironment (14). Anlotinib, a novel multi-targeted anti-angiogenic agent that primarily targets VEGFR, EGFR(Epidermal Growth Factor Receptor), and PDGFR(Platelet-Derived Growth Factor), has been approved by the NMPA(National Medical Products Administration) for the third-line treatment of advanced NSCLC. Sintilimab has been approved by the NMPA for first-line indications in two major populations: locally advanced or metastatic nonsquamous and squamous NSCLC patients. The PET-CT examination after 8 cycles of treatment showed that the lesion in the right lung was smaller than before, but the lesion in the left adrenal gland was larger than before. We believe that the enlargement of the tumor was caused by inflammatory cell infiltration, edema and necrosis in the tumor during immunotherapy, rather than true tumor proliferation. The pseudoprogression can also cause high 18F-FDG uptake. However, the patient’s symptoms were relieved and no new lesions were found. These proved that the treatments were effective. The effectiveness of the treatment was also confirmed by follow-up for nearly 3 years.

To the best of our knowledge, this is the first report showing a favorable response to sintilimab combined with anlotinib in PSC. Immunotherapy combined with the tyrosine kinase inhibitor anlotinib may be a promising strategy for treating patients with PSC. Further studies with more cases are warranted.

## Conclusion

PSC is a rare type of lung malignant tumor, and treatment options for this disease are limited. For some patients with advanced PSC, sintilimab combined with anlotinib treatment may result in a favorable outcome.

## Data availability statement

The original contributions presented in the study are included in the article/supplementary material. Further inquiries can be directed to the corresponding author.

## Ethics statement

This case report was approved by the Ethics Board of the Chengdu Fifth People’s Hospital, Chengdu, China. Written informed consent was obtained from the patient.

## Author contributions

Concept – GD, LH; Planning and Design – GD, LH; Supervision – LH; Materials – YL, QY, YH, BL, GW; Data Collection – YL, YH, BL, GW; Literature Review – GD. All authors contributed to the article and approved the submitted version.

## References

[1] Karim NA Schuster J Eldessouki I Gaber O Namad T Wang J. Pulmonary sarcomatoid carcinoma: university of Cincinnati experience. Oncotarget (2018) 9(3):4102–8. doi: 10.18632/oncotarget.234682 PMC5790524 29423107

[2] Steuer CE Behera M Liu Y Fu C Gillespie TW Saba NF. Pulmonary sarcomatoid carcinoma: an analysis of the national cancer data base. Clin Lung Cancer (2017) 18(3):286–92. doi: 10.1016/j.cllc.2016.11.016 28043773

[3] WHO Classification of Tumours Editorial Board. WHO classification of tumours. In: Thoracic tumours, 5th edition. Lyon: IARC (2021).

[4] Yendamuri S Caty L Pine M Adem S Bogner P Miller A. Outcomes of sarco matoid carcinoma of the lung: a surveillance, epidemiol ogy, and end results database analysis. Surgery (2012) 152(3):397–402. doi: 10.1016/j.surg.2012.05.007 22739072

[5] Lin Y Yang H Cai Q Wang D Rao H Lin S. Characteristics and prognostic analysis of 69 patients with pulmonary sarcomatoid carcinoma. Am J Clin Oncol (2016) 39(3):215–22. doi: 10.1097/COC.0000000000000101 25068469

[6] Paik PK Drilon A Fan P-D Yu H Rekhtman N Ginsberg MS. Response to MET inhibitors in patients with stage IV lung adenocarcinomas harboring MET mutations causing exon 14 skipping. Cancer Discovery (2015) 5(8):842–9. doi: 10.1158/2159-8290.CD-14-1467 PMC4658654 25971939

[7] Vieira T Girard N Ung M Monnet I Cazes A Bonnette P. Efficacy of first - line chemotherapy in patients with advanced lung sarcomatoid carcinoma. J Thorac Oncol (2013) 8(12):1574–7. doi: 10.1097/01.JTO.0000437008.00554.90 24389441

[8] Saffroy R Fallet V Girard N Mazieres J Sibilot DM Lantuejoul S. MET exon 14 mutations as targets in routine molecular analysis of primary sarcomatoid carcinoma of the lung. Oncotarget (2017) 8(26):42428–42437. doi: 10.18632/oncotarget.16403 28418914 PMC5522077

[9] Skoulidis F Goldberg ME Greenawalt DM Hellmann MD Awad MM Gainor JF. STK11/LKB1 mutations and PD-1 inhibitor resistance in KRAS-mutant lung adenocarcinoma. Cancer Discov (2018) 8:822–35. doi: 10.1158/2159-8290.CD-18-0099 PMC6030433 29773717

[10] Schrock AB Li SD Frampton GM Suh J Braun E Mehra R. Pulmonary sarcomatoid carcinomas commonly harbor either potentially targetable genomic alterations or high tumor mutational burden as observed by comprehensive genomic profiling. J Thorac Oncol (2017) 12(6):932–42. doi: 10.1016/j.jtho.2017.03.005 28315738

[11] Liu X Jia Y Stoopler MB Shen Y Cheng H Chen J. Next-generation sequencing of pulmonary sarcomatoid carcinoma reveals high frequency of actionable MET gene mutations. J Clin Oncol (2015) 34(8):794–802. doi: 10.1200/JCO.2015.62.0674 26215952

[12] Vieira T Antoine M Hamard C Fallet V Duruisseaux M Rabbe N. Sarcomatoid lung carcinomas show high levels of programmed death ligand-1 (PD-L1) and strong immune-cell infiltration by TCD3 cells and macrophages. Lung Cancer Amst Neth. (2016) 98:51–8. doi: 10.1016/j.lungcan.2016.05.013 27393506

[13] Domblides C Leroy K Monnet I Mazières J Barlesi F Gounant V. Efficacy of immune checkpoint inhibitors in lung sarcomatoid carcinoma. J Thorac Oncol (2020) 12(6):932–2. doi: 10.1016/j.jtho.2020.01.014 31991225

[14] Liang H Wang M. Prospect of immunotherapy combined with anti-angiogenic agents in patients with advanced non-small cell lung cancer. Cancer Manag Res (2020) 11:7707–19. doi: 10.2147/CMAR.S212238 PMC6699593 31616186

